# Supplementary material for: Integrating genomic structural equation modeling and experimental validation to unravel the genetic basis of male genital lichen sclerosus
Source: Front Immunol. 2026 Jul 16;17:1857945. doi: 10.3389/fimmu.2026.1857945 (PMC13435250; doi:10.3389/fimmu.2026.1857945)
Supplement: Supplementary file 6 [file SupplementaryFile5.zip › result/fuma_PSYCH_factor_sCCA3_focus.chr6.chr6.160580497.162169564.pop1.pdf]

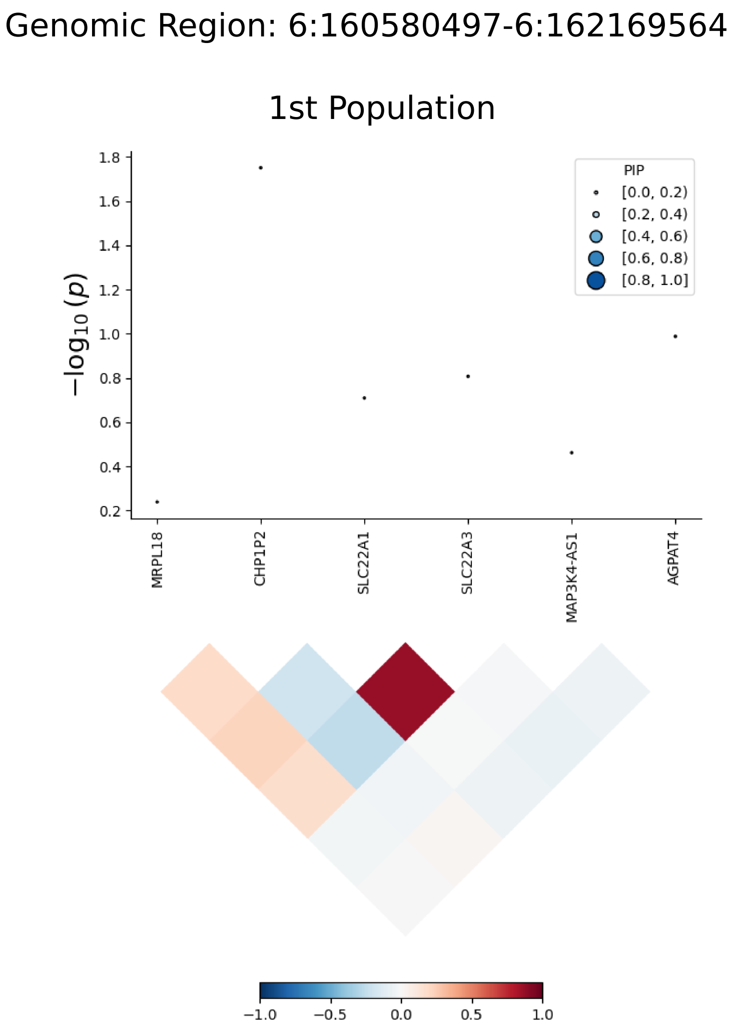

Genomic Region: 6:160580497-6:162169564
1st Population
$-\log_{10}(p)$
1.8
1.6
1.4
1.2
1.0
0.8
0.6
0.4
0.2
PIP
[0.0, 0.2)
[0.2, 0.4)
[0.4, 0.6)
[0.6, 0.8)
[0.8, 1.0]
MRPL18
CHP1P2
SLC22A1
SLC22A3
MAP3K4-AS1
AGPAT4
−1.0
−0.5
0.0
0.5
1.0
